# Deficient responses from the lateral geniculate nucleus in humans with amblyopia

**DOI:** 10.1111/j.1460-9568.2009.06650.x

**Published:** 2009-03

**Authors:** Robert F Hess, Benjamin Thompson, Glen Gole, Kathy T Mullen

**Affiliations:** 1 McGill Vision Research (H4.14), Department of Ophthalmology, McGill University687 Pine Avenue West, Montreal, QC H3A 1A1, Canada; 2 The Wesley Hospital Research Institute, Department of Ophthalmology, University of QueenslandBrisbane, Qld, Australia

**Keywords:** amblyopia, functional magnetic resonance imaging, lateral geniculate nucleus, vision

## Abstract

Amblyopia or lazy eye is the most common cause of uniocular blindness in adults. It is caused by a disruption to normal visual development as a consequence of unmatched inputs from the two eyes in early life, arising from a turned eye (strabismus), unequal refractive error (anisometropia) or form deprivation (e.g. cataract). Animal models based on extracellular recordings in anesthetized animals suggest that the earliest site of the anomaly in the primate visual pathway is the primary visual cortex (corresponding to the striate cortex, cytoarchitectonic area 17 and area V1), which is where inputs from the two eyes are first combined in an excitatory fashion, whereas more distal and monocular processing structures such as the retina and lateral geniculate nucleus (LGN) are normal. Using high-field functional magnetic resonance imaging in a group of human adults with amblyopia, we demonstrate that functional deficits are first observable at a thalamic level, that of the LGN. Our results suggest the need to re-evaluate the current models of amblyopia that are based on the assumption of a purely cortical dysfunction, as well as the role for the LGN in visual development.

## Introduction

Amblyopia (incidence 3%) is a disorder of human visual development that produces a uniocular visual loss, in which individuals have an impaired amblyopic eye and a normal fixing eye. The physiological origins of the deficit have been extensively investigated using single-cell neurophysiology in animal models with amblyopia produced artificially using a surgically induced strabismus, optically induced ansiometropia, or from deprivation by monocular lid suture. The current consensus is that the functions of the retina (Cleland *et al.*, 1980, 1982) and lateral geniculate nucleus (LGN) (Derrington & Hawken, 1981; Blakemore & Vital-Durand, 1986; Sasaki *et al.*, 1998; Levitt *et al.*, 2001) are normal (but see Sherman *et al.*, 1975; Ikeda & Tremain, 1978; Chino *et al.*, 1994; Yin *et al.*, 1997; Levitt *et al.*, 2001) although the LGN layers that receive input from the affected eye display histological abnormalities (Guillery, 1972; Einon *et al.*, 1978; Tremain & Ikeda, 1982; von Noorden & Crawford, 1992). Anomalous neural responses are first found in layer 4c of the striate cortex (cytoarchitectonic area 17 in cat and area V1 in primate) and the currently accepted notion that the earliest site of amblyopia is in the input layers of the striate visual cortex is based primarily on there being very little binocular excitatory convergence at the LGN level. In human amblyopia less invasive techniques have to be used but these have supported a cortical origin. Retinal evoked potentials suggest that the eye itself is normal (Hess & Baker, 1984; Hess *et al.*, 1985) and functional magnetic resonance imaging (fMRI) demonstrates a cortical deficit with reduced activations in V1 and extrastriate cortex (Barnes *et al.*, 2001; Muckli *et al.*, 2006; Li *et al.*, 2007).

Our understanding of the function of the LGN in vision has undergone important changes in recent years (Sherman & Guillery, 2002). Its role as a simple relay station for retinal information has been replaced by a realization that ascending information from the retina undergoes complex modification within the LGN by inputs emanating from the cortex, brainstem and hypothalamus (Sherman & Guillery, 1998). Only a fraction (approximately 6%) of the cells in the LGN are concerned with feedforward transmission from the retina (termed ‘drivers’), with the vast majority modifying these afferent signals (termed ‘modulators’) (Sherman & Guillery, 2002). Neurophysiological studies in anesthetized animals are biased towards the LGN ‘driver activity’ as ‘modulator activity’ is thought to be more susceptible to anesthesia. This may explain why the histological abnormalities in the LGN layers receiving input from the deprived eye (Guillery, 1972; Einon *et al.*, 1978; Tremain & Ikeda, 1982; von Noorden & Crawford, 1992) have not been found previously to have accompanying functional correlates (Derrington & Hawken, 1981; Blakemore & Vital-Durand, 1986; Sasaki *et al.*, 1998; Levitt *et al.*, 2001).

Functional magnetic resonance imaging provides a unique opportunity to address LGN function in humans. It avoids the confounding problems of anesthesia and, by including the activation of local field potentials rather than just spiking activity (Logothetis *et al.*, 2001), assesses the modulator-based activation of the majority LGN cells. LGN functional imaging has been constrained by technical limitations imposed by its small size and low signal strength; however, the normal LGN can now be localized and its responses to achromatic (Chen *et al.*, 1998a,b, 1999; Fujita *et al.*, 2001; Kastner *et al.*, 2004; Schneider *et al.*, 2004) and chromatic (Mullen *et al.*, 2008) stimuli quantified. Here we use high-field strength fMRI to assess the functional integrity of the LGN in human amblyopia by comparing LGN activation when driven by the amblyopic and fellow fixing eyes, using broadband stimuli with chromatic and achromatic contrast to include responses in the parvocellular, magnocellular and koniocellular LGN layers.

## Materials and methods

### Subjects and stimuli

We studied the responses in six amblyopes selected to cover a range of etiologies (three strabismic, one anisometropic and two form-deprivation amblyopes), as detailed in Table 1. We measured the region of the retina used for fixation in all subjects using visuoscopy (Table 1) and monitored the fixation eye movements of all amblyopic subjects while they were viewing the stimulus in a control experiment run outside the scanner using an in-house video monitoring of the pupil with subsequent off-line analysis of fixational variability. All subjects fixated on the central fixation mark provided, the amblyopic eye being more unsteady than the fellow fixing eye (Table 1). The degree of unsteadiness was small compared with the field size used (12°). All experiments were undertaken with the understanding and written consent of each subject, and the study conformed to The Code of Ethics of the World Medical Association (Declaration of Helsinki), printed in the *British Medical Journal* (18 July 1964) and the Ethics board of the Queensland Institute of Technology.

**Table 1:** Clinical details for the six amblyopic participants

Subject/type of amblyopia	Refraction	Acuity	Eye alignment	Fixation centration	Fixation variance	History
JLK/strabismic	+0.75D +0.765D	6/5 6/48	LET	2° eccentric	±0.74° ±2.7°	Large LET patching age 2 years, surgery age 5 years
BB/strabismic	+0.50/−0.5 × 160 +1.00/−0.25 × 180	6/5 6/600	LET	Central	±0.39° ±0.52°	Surgery to correct large angle eso age 7 years
CRF/strabismic	−2.75D −3.00D	6/6 6/240	LXT, L hypoT	4° eccentric	±0.10° ±0.39°	LET and surgery in infancy and age 25 years
SJH/anisometrope	+7.00/−3.00 × 150 +2.50/−1.25 × 80	6/30 6/4.5	Ortho	Central	±0.38° ±0.35°	First Rx age 19 years
DJL/deprivation	+8.25/−1.00 × 90 +0.25D	CF 6/6	RET	6° eccentric	±3.1° ±0.18°	Two operations for ET age 9 years
MLTdeprivation	−2.25D −1.50D	6/6 CF	LXT	2° eccentric	±0.42° ±1.8°	Cataract surgery age 7 years

R, right eye; L, left eye; ET, esotropia; XT, exotropia; ortho, orthotropic alignment; D, dioptre sphere; CF, count fingers; eso, esotropia; RX, refractive correction; hypoT, hypotropia.

The stimulus was a high-contrast squarewave checkerboard stimulus (check size, 1.5°; field size, 12° width × 10° height) with both AC and DC chromatic and achromatic squarewave modulation at 16 Hz, used to provide sufficient stimulation to activate the chromatic and achromatic cells of the magnocellular, parvocellular and koniocellular layers of the LGN (Mullen *et al.*, 2008). We referenced responses to a blank with very low luminance as most of the cells in the LGN are not DC-balanced and respond to a mean light level. A central black fixation dot was provided throughout all presentations.

### Experimental protocols

A standard block design was used composed of alternate stimulus and blank intervals (18 s stimulation, 18 s fixation, 10 blocks per run). Each stimulus was presented in a two alternate forced choice paradigm within a 3 s cycle; each stimulus presentation was for 800 ms with an interstimulus interval of 200 ms and 1.2 s for response. To control for attentional modulation known to affect cortical and subcortical structures (O’Connor *et al.*, 2002), subjects performed a two alternate forced choice contrast discrimination task that involved discriminating subtle changes in the contrast of pairs of alternately presented stimuli within a stimulus cycle and responding with a button press. During the fixation epochs dummy button presses were made. The contrast difference between alternately presented stimuli was detectable with all subjects performing the task with an average performance of 98.5 ± 2% with the amblyopic eye and 97.8 ± 2% with the fellow fixing eye, demonstrating that the targets were visible to each eye and properly imaged on their retinas. During the experimental paradigm participants viewed the stimuli monocularly and a tight-fitting eye patch was used to occlude one eye.

### Magnetic resonance imaging

All magnetic resonance images were acquired using a 4T Bruker MedSpec system at the Centre for Magnetic Resonance (Brisbane, Australia). A transverse electromagnetic head coil was used for radiofrequency transmission and reception (Vaughan *et al.*, 2002). For the fMRI experimental study, 256 T2*-weighted gradient-echo echoplanar images depicting blood oxygen level-dependent (BOLD) contrast (Ogawa *et al.*, 1990) were acquired in each of 24 planes with TE 30 ms, repetition time (TR) 1500 ms, in-plane resolution 3.1 × 3.1 mm and slice thickness 3 mm (0 mm gap). The slices were taken parallel to the calcarine sulcus and arranged to include the anatomical location of the LGN. Two to three fMRI scans were performed in each session. Head movement was limited by foam padding within the head coil. In the same session, a high-resolution three-dimensional T1 image was acquired using an MP-RAGE sequence with TI 1500 ms, TR 2500 ms, TE 3.83 ms and a resolution of 0.9 mm^3^.

### Lateral geniculate nucleus localization

The LGN localization data were acquired in a separate scanning session conducted under binocular viewing conditions. Left and right LGNs were localized in each participant using both anatomical and functional data. During scanning, participants viewed alternating blocks of the high-contrast squarewave checkerboard (see ‘Subjects and stimuli’ section above) and the blank intervals with a small dim fixation dot. Each block lasted 18 s and was repeated 10 times in each of two scanning runs. Data were analysed for each individual participant using a general linear model analysis and statistical maps of t-values were visualized at the false discovery rate corrected (Benjamini & Hochberg, 1995) level of *q* < 0.001. LGNs were defined as a stimulus responsive region in the appropriate anatomical location (Kastner *et al.*, 2004). Regions of interest were created by first identifying the peak voxel (i.e. the voxel whose activity was most reliably correlated with the presentation of the stimulus) within the LGN region, then a cube of 10 mm^3^ was centered on the peak voxel and the region of interest was defined as all voxels within the cube contiguous with the peak voxel whose activity in response to the checkerboard stimulus was above threshold. The Talairach coordinates of all of the LGNs are given in Table 2.

**Table 2:** The LGN coordinates and volumes located in stereotaxic space for the six subjects

	Left LGN				Right LGN			
	Coordinates (mm)*				Coordinates (mm)*			
Subject	*x*	*y*	*z*	Volume (mm^3^)	*x*	*y*	*z*	Volume (mm^3^)
BB	−23	−26	−3	155	25	−25	2	86
CRF	−21	−27	−3	905	20	−27	−1	852
DJL	−19	−27	−3	91	18	−27	−1	147
JLK	−21	−24	−3	542	23	−22	−2	612
SJH	−23	−22	−4	243	25	−24	−2	283
MLT	−19	−28	1	518	20	−27	0	655
Mean	−21	−26	−2	409	22	−25	−1	439
SD	2	2	2	306	3	2	1	310

* Talairach coordinates (Talairach & Tournoux, 1988).

### Data analysis

Data analysis was conducted with the commercially available Brain Voyager analysis package (version 1.9.10, Brain Innovations, Maastricht, The Netherlands). Functional scans were high-pass filtered and motion corrected using subroutines within Brain Voyager. They were then aligned to each subject’s high-resolution anatomical images (resampled at 1 mm^3^) and transformed to Talairach space (Talairach & Tournoux, 1988). Time series data were extracted from the LGN region of interest for each individual participant using an event-related averaging paradigm. Time series data for each stimulation event were normalized to the directly preceding 2TRs, when the subject was viewing the blank, to provide a baseline for the %BOLD change measure. Peak %BOLD response was calculated as the maximum %BOLD change at a time-point within a window starting 4TRs (6 s) after the onset of the stimulus and ending 4TRs after the offset of the stimulus. Average %BOLD change was calculated as the average %BOLD values present within the same window used for peak %BOLD calculations. Two scanning runs were conducted per eye and time series data were combined for subsequent extraction of %BOLD change data from the LGN region of interest.

## Results

**Fig. 1:**
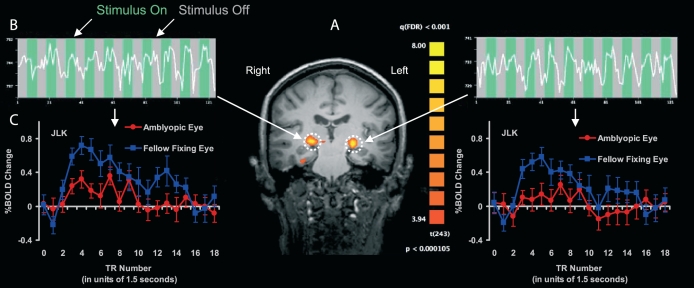
(A) A stereotaxic representation (coronal slice, radiological convention) for one subject (JLK), a strabismic amblyope, showing the localization of the left and right LGNs. The two LGNs are indicated by the white dashed circles for illustration purposes only. The activation depicted is the result of a general linear model analysis contrasting binocular stimulation with fixation. False discovery rate (FDR) corrected for multiple comparisons (*q* < 0.001) was used to define regions of interest (ROIs) for each subject. The ROI was never greater than 10 mm^3^ and was smaller than the region of activation shown here. The color bar represents the *t*-values. (B) The ROI time series for the left and right LGN obtained from our block design. Vertical colored stripes indicate the blocks of stimulation (green) and non-stimulation (grey) epochs (each lasting 12 TRs or 18 s), and the white line indicates the raw time-course in units of voxel intensity over time (in units of 1 TR or 1.5 s). The time-course clearly follows the stimulation epochs indicated in the panels. (C) The two panels in the lower left and right corners of the figure depict %BOLD change as a function of time for the right and left LGNs, respectively, when driven by either the fellow (blue line) or amblyopic (red line) eye. %BOLD signal change was calculated on a point-by-point basis by normalizing each time-point within the stimulation epoch to the average of the last six TRs in the fixation epoch. Stimulus onset was at TR no. 0 and stimulus offset was at TR no. 12. The responses are averaged across scans. Error bars represent ± 1 SEM. Results show that there is reduced activation in each LGN when driven by the amblyopic eye compared with the fellow fixing eye.

**Fig. 2:**
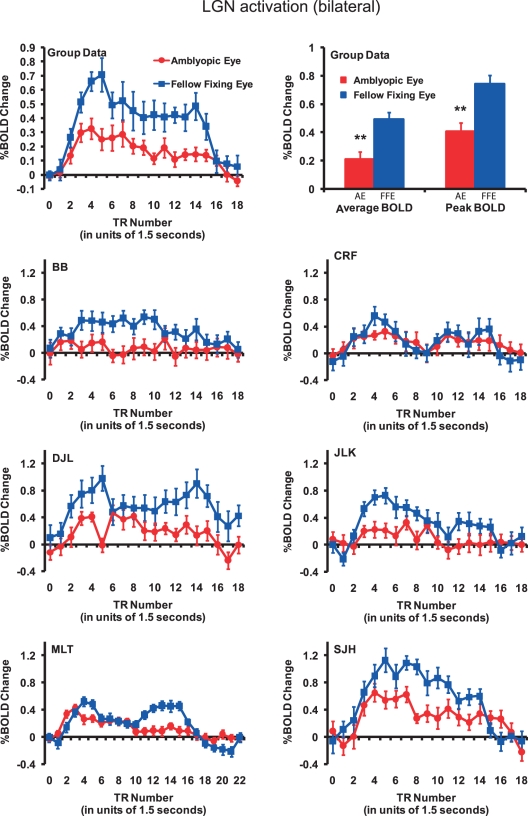
Group (top left) and individual (lower three rows) data showing LGN activation time-courses for six human amblyopes averaged across left and right LGNs comparing stimulation of fixing and amblyopic eyes (for details of calculations see Fig. 1). Top right panel shows two measures of the activation derived from the group data. The average %BOLD response (as marked) was calculated by averaging the 12 time-points (TRs) starting from TR no. 4 (6 s after stimulus onset). The peak %BOLD change was calculated as the maximum %BOLD change value that occurred within the same time window. Amblyopes as a group showed significantly (**) reduced average and peak LGN activation for amblyopic compared with fellow fixing eye stimulation [average BOLD: *t*(5) = 4.79, *P* < 0.005, two-tailed; peak BOLD: *t*(5) = 5.25, *P* = 0.003, two-tailed]. Error bars show ± 1 SEM. **P < 0.01.

Figure 1 shows the localization of the LGNs in one subject (JLK, a strabismic amblyope), a time series response for our 10 mm^3^ region of interest from each LGN illustrating our block design, and plots of the averaged activation comparing left and right eye stimulation for each LGN. The responses from each LGN were clearly reduced when driven by the amblyopic eye (red symbols) compared with the normal fixing eye (blue symbols). The results in Fig. 2 (lower six panels) show LGN activations for each individual amblyope comparing stimulation by the fixing and amblyopic eye. Results are averaged across both LGNs of each subject. In all cases, with the possible exception of CRF, there was less LGN activation when driven by the amblyopic eye (red symbols). The top row of Fig. 2 shows the averaged group data for all six subjects (12 LGNs) from which we have derived two measures for both fixing and amblyopic eye stimulation, i.e. the averaged activity across the time series and the peak activity. There was a significant difference between amblyopic and fixing eye activations, with the LGN activation reduced when it was driven by the amblyopic eye [peak BOLD: *t*(5) = 5.25, *P* = 0.003; average BOLD: *t*(5) = 4.79, *P* = 0.005, two-tailed]. There was no significant correlation between either of these measures and the visual acuity of the amblyopic eye (peak BOLD: Spearman’s rho = −2.32, *P* = 0.67; average BOLD: Spearman’s rho = −2.32, *P* = 0.67).

Similar reductions in BOLD activation were obtained when evaluating the responses of individual LGNs. Rather than comparing left and right LGNs for each subject, we compared the LGNs contralateral and ipsilateral to the amblyopic eye for each subject (shown in supplementary Figs 1S and 2S, respectively). The LGN contralateral to the amblyopic eye draws on nasal retinal fibers from the amblyopic eye, whereas the ipsilateral LGN draws on temporal retinal fibers from the amblyopic eye. For stimulation of the normal fixing eye we found no difference between contralateral and ipsilateral LGN activation, indicating no difference between nasal and temporal retinal inputs to LGN. Such naso-temporal differences have been reported at the cortical level (V1) using fMRI (Toosy *et al.*, 2001). For amblyopic eye stimulation we also found no significant differences between activations of contralateral LGN (inputs from nasal fibers) and ipsilateral LGN (temporal fiber inputs). Large eccentric fixations in the amblyopic eyes would create asymmetries in the proportions of the nasal and temporal retina stimulated compared with a centrally fixated stimulus. This would be expected to create asymmetries in the activations of the two LGNs, i.e. between contralateral (nasal) and ipsilateral (temporal) inputs to the LGNs. The fact that there were no such contralateral vs. ipsilateral asymmetries in the LGN activation, especially in the amblyopes with some eccentric fixation (DJL, BB, JLK and CRF), indicated that any eccentric fixation by the amblyopes was not responsible for the results that we report. Moreover, our main significant effect in Fig. 2 was based on the average of both LGNs of each subject, in which any differences in the proportion of nasal to temporal field stimulated have been cancelled out.

## Discussion

This is the first quantitative analysis of the function of the LGN in human amblyopia. Our finding of a functional deficit in the LGN of amblyopes with very different etiologies is novel and shows that the amblyopic deficit is not confined to the cortex as is currently believed. Our current psychophysical and neurophysiological models of amblyopia are predicated on the assumption that the loss is of purely cortical origin, these will now need to be re-evaluated in the light of these findings. Moreover, the loss of function in the LGN, whose neurons are monocular, arising from a developmental disorder normally associated with a mismatch in binocular activation, indicates that the LGN plays a more important and complex role in early visual development than previously thought. Our results are consistent with a previous informal clinical report from a single anisometropic amblyope in which it was noted that the LGN, when driven by the amblyopic eye, was more difficult to image (Miki *et al.*, 2003). Although there was no detailed numeric analysis of the response activations, this clinical observation is consistent with our quantitative results.

There are a number of possible explanations for our finding. It is unlikely that either the degree of eccentric fixation or the fixation instability can account for the reduction in response that we observe. As argued in the Results, eccentric fixations would be expected to produce asymmetries in the contralateral vs. ipsilateral LGN activations, whereas none are found. Moreover, our results for the average of both LGNs cancel out the effects of any asymmetry, and still show a significant deficit. As we used a relatively large field size (12° across), eccentric fixation or any fixation instability would have to be very large for subjects to completely miss the test stimulus or for a large asymmetry in the stimulation of the nasal vs. temporal fields to be created. Moreover, our calculations show that there are no significant correlations in our data between the differences in LGN activation and either the degree of fixation variability or the degree of eccentric fixation. We thus conclude that the deficit in LGN function that we find for inputs from the amblyopic eye represents a genuine physiological disorder in the LGN in amblyopia.

One question that arises is whether the reduced LGN response could be due to reduced retinal function arising from the amblyopic eye. Previously published data suggesting that the amblyopic eye is normal are quite comprehensive and involve single-cell recording in deprived animals (Cleland *et al.*, 1980, 1982; Crewther *et al.*, 1985), evoked potential measurements in human amblyopes (Hess & Baker, 1984; Hess *et al.*, 1985), and clinical measurements of optic nerve fibre density in human amblyopes (Bozkurt *et al.*, 2003). Therefore, the deficit reported here probably reflects LGN function *per se* or an anomaly in the corticogeniculate processing of information. Animal models of amblyopia have provided evidence for morphological changes in the LGN, for example in neurons in the layers of the binocular segment of the LGN that receive input from the deprived eye in animals (Guillery, 1972; Tremain & Ikeda, 1982), and are supported by a postmortem study in human (von Noorden *et al.*, 1983; von Noorden & Crawford, 1992). There are also some isolated reports of functional abnormalities in the cellular responses from the LGN in cats deprived of vision or eye misalignment including a selective loss of X-cell function (Ikeda & Wright, 1976; Ikeda *et al.*, 1978; Chino *et al.*, 1994), a selective loss of Y-cells (Sherman *et al.*, 1975; Yin *et al.*, 1997), and more subtle changes in responsiveness (Levitt *et al.*, 2001). Our results indicate that these changes may have functional correlates in the LGN in human amblyopia.

A more recent analysis of thalamic processing has identified at least two separate processes: ‘drivers’ and ‘modulators’ (Sherman & Guillery, 1996, 1998, 2002; Reichova & Sherman, 2004). ‘Drivers’ refer to neurons that transmit the information to be relayed from sensory end organs, whereas ‘modulators’ serve to modulate the thalamic transmission of the driver input. As fMRI activations reflect local field potentials more than all-or-nothing action potentials (Logothetis *et al.*, 2001) and as only approximately 6% of LGN synaptic junctions subserve driver-based activity (Sherman & Guillery, 1998), it seems likely that the reduced fMRI activation reported here during amblyopic eye stimulation reflects a change to the modulatory control of the LGN, mostly coming from layer 6 of V1 (Van Horn & Sherman, 2004).

Our conclusion that amblyopia can no longer be regarded as an exclusively cortical disorder has important implications for our understanding of how binocular competition regulates brain plasticity during early visual development. The cortex, which has long been considered the site of amblyopia (Derrington & Hawken, 1981; Blakemore & Vital-Durand, 1986; Crewther & Crewther, 1990), is where the inputs from the two eyes first combine and compete for representation. Although the unique laminar structure of the LGN and its monocular neurons keep the inputs of the two eyes separate, it also offers an excellent site for the descending regulation of the monocular input from the amblyopic eye. The corticogeniculate feedback from layer 6 of the cortex innervates LGN relay cells directly and also indirectly via the thalamic reticular nucleus, leading to a direct excitation via the former and a disynaptic inhibition via the latter. This forms part of the proposed modulator circuit and is subserved by the vast majority of synaptic junctions in the LGN (Sherman & Guillery, 1998, 2002). It is unclear whether the reduced LGN activation in adult amblyopes is due to local inhibitory influences within the LGN or the result of feedback influences from the cortex. The corticogeniculate feedback pathway (Tsumoto & Freeman, 1981; Freeman & Tsumoto, 1983) may underlie the inhibitory binocular interactions in the LGN of normal animals (Sanderson *et al.*, 1969, 1971; Rodieck & Dreher, 1979; Pape & Eysel, 1986; Xue *et al.*, 1987). Furthermore, there is evidence that the loss of cortical binocularity in deprived cats is due to a GABA-mediated inhibition (Duffy *et al.*, 1976; Burchfiel & Duffy, 1981; Sillito *et al.*, 1981; Mower *et al.*, 1984) that may also modulate the function of the LGN via corticogeniculate feedback.

## Abbreviations

BOLD: blood oxygen level-dependent
fMRI: functional magnetic resonance imaging
LGN: lateral geniculate nucleus
TR: repetition time

## Supporting information

Fig. S1. Group and individual time series data for activation of the LGN contralateral to the amblyopic eye.

Fig. S2. Group and individual time series data for activation of the LGN contralateral to the amblyopic eye.
