# Supplementary material for: Deficient responses from the lateral geniculate nucleus in humans with amblyopia
Source: Eur J Neurosci. 2009 Mar;29(5):1064–70. doi: 10.1111/j.1460-9568.2009.06650.x (PMC2695153; doi:10.1111/j.1460-9568.2009.06650.x)
Supplement: Supplementary file 1 [file ejn0029-1064-SD1.doc]

**Fig. S1**. Group (top left) and individual (lower 3 rows) time series data for activation of the LGN contralateral to the amblyopic eye. When stimulated by the amblyopic eye nasal retinal fibres dominate the activation of the contralateral LGN. On average, the LGN activation by the amblyopic eye is significantly reduced compared to activation by the fellow fixing eye for both peak and average time course measures: average BOLD t(5) = 2.94, *P* = 0.016, peak BOLD t(5) = 3.45, *P* = 0.009, 1-tailed, with a Bonferroni correction for multiple comparisons for contra- and ipsilateral LGN (Figure 2S), *P*= 0.025. Error bars show ±1 SEM. *P* < 0.005.
