# Supplementary material for: Deficient responses from the lateral geniculate nucleus in humans with amblyopia
Source: Eur J Neurosci. 2009 Mar;29(5):1064–70. doi: 10.1111/j.1460-9568.2009.06650.x (PMC2695153; doi:10.1111/j.1460-9568.2009.06650.x)
Supplement: Supplementary file 2 [file ejn0029-1064-SD2.doc]

**Fig. S2**. Group (top left) and individual (lower 3 rows) time series data for activation of the LGN ipsilateral to the amblyopic eye. When stimulated by the amblyopic eye temporal retinal fibres dominate the activation of the ipsilateral LGN. On average, the LGN activation by the amblyopic eye is significantly reduced compared to activation by the fellow fixing eye for both peak and average time course measures: average BOLD t(5) = 8.02, *P* < 0.0005, peak BOLD t(5) = 7.57, *P* = 0.001, 1-tailed, with a Bonferroni correction for multiple comparisons for contra- (Figure 1S) and ipsilateral LGN, *P* = 0.025. Error bars show ±1 SEM. *P* < 0.001.
